# Parental Overcontrol and Its Role in the Development of Anorexia Nervosa: A Case Report

**DOI:** 10.1192/j.eurpsy.2025.1395

**Published:** 2025-08-26

**Authors:** L. Demiraj, E. Sotiri

**Affiliations:** 1 Psychiatry, UHC “Mother Teresa”, Tirana, Albania

## Abstract

**Introduction:**

Anorexia nervosa is a complex eating disorder often associated with various psychological factors, including maladaptive coping mechanisms and dysfunctional family dynamics. This case report illustrates the impact of parental overcontrol on the development and persistence of anorexia nervosa in a young female patient.

**Objectives:**

To present the case of a 19-year-old female patient with anorexia nervosa, emphasizing the influence of her childhood experiences, particularly parental overcontrol, on her eating behaviors and psychological state.

**Methods:**

Case report.

**Results:**

The patient presented to the emergency room accompanied by family members due to extreme weight loss (29.6 kg; BMI 11.4 kg/m²) and severe purging behaviors, including self-induced vomiting and the consumption of 400 laxative tablets daily for six months. Her history of mental health issues began in 2013, characterized by severe anxiety related to separation from her parents and academic performance, culminating in a suicide attempt. In 2019, she developed purging behaviors and received outpatient treatment for eight months, which she discontinued. Symptoms resumed, and after reaching a target weight of 30 kg, she set a new target weight of 20 kg. The patient’s behavior was influenced by insecure attachment modeled by her father, who exhibited controlling and intrusive parenting styles.

A review study (Gale C.J., et al. A review of the father-child relationship in the development and maintenance of adolescent anorexia and bulimia nervosa. *Issues Compr. Pediatr. Nurs.* 2013;36:48–69. doi: 10.3109/01460862.2013.779764.) investigated the role of the father–child relationship as a risk or maintenance factor of Eating Disorders in developmental age. Results showed that within the relationship of the father and child, and especially with daughters, there are several themes such as conflict and communication, parental protection and psychological control, emotional regulation and self-esteem, and self-perfectionism that appear to condition the child’s level of self-determining autonomy and can consequently impact maladaptive eating attitudes and psychopathology. (Criscuolo, M., et al (2023). Parental Emotional Availability and Family Functioning in Adolescent Anorexia Nervosa Subtypes. *International Journal of Environmental Research and Public Health*, 20(1), 68. https://doi.org/10.3390/ijerph20010068)

**Conclusions:**

This case highlights the significant association between parental overcontrol and the development of anorexia nervosa, illustrating the need for targeted interventions that address both psychological and familial factors contributing to the disorder. Further discussion is warranted on the implications of parenting styles in the management and prevention of eating disorders.

**Disclosure of Interest:**

None Declared

